# APC/C-Mediated Degradation of dsRNA-Binding Protein 4 (DRB4) Involved in RNA Silencing

**DOI:** 10.1371/journal.pone.0035173

**Published:** 2012-04-24

**Authors:** Katia Marrocco, Marie-Claire Criqui, Jérôme Zervudacki, Gregory Schott, Herfried Eisler, Aude Parnet, Patrice Dunoyer, Pascal Genschik

**Affiliations:** 1 Institut de Biologie Moléculaire des Plantes, Centre National de la Recherche Scientifique, Unité Propre de Recherche 2357, Conventionné avec l'Université de Strasbourg, Strasbourg, France; 2 Swiss Federal Institute of Technology (ETH), Zurich, Switzerland; Instituto de Biología Molecular y Celular de Plantas, Spain

## Abstract

**Background:**

Selective protein degradation via the ubiquitin-26S proteasome is a major mechanism underlying DNA replication and cell division in all Eukaryotes. In particular, the APC/C (Anaphase Promoting Complex or Cyclosome) is a master ubiquitin protein ligase (E3) that targets regulatory proteins for degradation allowing sister chromatid separation and exit from mitosis. Interestingly, recent work also indicates that the APC/C remains active in differentiated animal and plant cells. However, its role in post-mitotic cells remains elusive and only a few substrates have been characterized.

**Methodology/Principal Findings:**

In order to identify novel APC/C substrates, we performed a yeast two-hybrid screen using as the bait Arabidopsis APC10/DOC1, one core subunit of the APC/C, which is required for substrate recruitment. This screen identified DRB4, a double-stranded RNA binding protein involved in the biogenesis of different classes of small RNA (sRNA). This protein interaction was further confirmed *in vitro* and in plant cells. Moreover, APC10 interacts with DRB4 through the second dsRNA binding motif (dsRBD2) of DRB4, which is also required for its homodimerization and binding to its Dicer partner DCL4. We further showed that DRB4 protein accumulates when the proteasome is inactivated and, most importantly, we found that DRB4 stability depends on APC/C activity. Hence, depletion of *Arabidopsis* APC/C activity by RNAi leads to a strong accumulation of endogenous DRB4, far beyond its normal level of accumulation. However, we could not detect any defects in sRNA production in lines where DRB4 was overexpressed.

**Conclusions/Significance:**

Our work identified a first plant substrate of the APC/C, which is not a regulator of the cell cycle. Though we cannot exclude that APC/C-dependent degradation of DRB4 has some regulatory roles under specific growth conditions, our work rather points to a housekeeping function of APC/C in maintaining precise cellular-protein concentrations and homeostasis of DRB4.

## Introduction

The ubiquitin-26S proteasome system (UPS) is the major regulator to control the abundance of key factors and enzymes in all eukaryotes [1]. In higher plants, the UPS plays a central role in cell cycle regulation, hormone signalling, development, chromatin regulation or response to environmental stresses among others [2], [3]. Targets of the UPS are first poly-ubiquitylated by the sequential action of three enzymes (E1s, E2s and E3s) and then degraded by the 26S proteasome. The E3 enzymes (also called Ubiquitin protein Ligases) play the central role in this mechanism as they specifically recognise and select substrates. The Anaphase Promoting Complex/Cyclosome (APC/C) is a conserved multi-subunit E3 ligase, composed of at least 11 core subunits and a co-activator protein from the CDC20/FIZZY or CDH1/FIZZY-RELATED families [4], [5]. APC2 and APC11 constitute the catalytic module of the enzyme, whereas CDC20 and CDH1 have been shown to bind and recruit substrates. More recently, another subunit of the APC/C, APC10 has also been identified as a part of a catalytic module together with APC2 and CDH1 and to be directly involved in the substrate recognition step and poly-ubiquitin chain extension [6], [7], [8].

The APC/C is a key regulator of the cell cycle transitions that especially acts at the metaphase to anaphase transition and at the exit from mitosis [5]. During prometaphase, spindle-assembly-checkpoint proteins such as MAD2 and BUBR1 are activated at kinetochores and inhibit by sequestrating the APC/C^CDC20^. In metaphase, when all kinetochores are attached to microtubules, APC/C^CDC20^ becomes activated and promotes the degradation of the anaphase inhibitor PDS1/SECURIN and thereby activates the protease separase. Separase then cleaves cohesin complexes and initiates sister-chromatid separation. After anaphase, APC/C^CDH1^ mediates the final degradation of mitotic B-type cyclins which leads to Cyclin-Dependent Kinase 1 (CDK1) inactivation as well as many other cell cycle regulators such as Plk1, Aurora kinases, Tpx2, BUB1 or CDC20 among others and thus enables exit from mitosis [5]. Moreover during G1, the APC/C remains active and plays critical roles in maintaining G1 phase and controlling the onset of DNA replication, thus protecting chromosomal integrity [9].

Expression analysis of APC/C members in mammals has revealed that this complex is not only expressed in dividing cells [10]. Contrary to CDC20, CDH1 is also expressed in differentiated cells such as neurons. It has been shown that APC/C^CDH1^ drives cell differentiation in muscles through the degradation of Skp2 and Myf5 [11]. More surprisingly, APC/C has been shown to have a crucial role in post-mitotic neurons at different levels like axonal growth and patterning. SnoN and Id2 are two nuclear proteins identified as targets of the APC/C in these processes, as in CDH1-depleted neurons, both proteins are stabilized [9]. In Drosophila, APC/C acts at the pre-synaptic level controlling synaptic size by targeting Liprin-∝ for degradation [12]. Whereas in *C. elegans*, a recent study has shown that APC/C acts at the post-synaptic level controlling the number of glutamate receptor by targeting GluR1, a subunit of AMPA receptors, for degradation [13].

In *Arabidopsis*, all different subunits of APC/C have been identified [14] and several studies support a key role of APC/C in the regulation of cell cycle [3]. Mutations in different subunits of APC/C led to female gametogenesis defects (typically at the first mitosis) presumably due to the inability to degrade mitotic cyclins. In addition, the *APC3b* subunit has also been implicated in postembryonic differentiation at the meristems [15], whereas *CCS52A*, plant *CDH1* orthologues, have been shown to control endoreduplication in rosette leaves and meristem maintenance in roots [16], [17]. So far, only substrates of APC/C that regulates the cell cycle progression have been identified in plants. However, we have shown in a previous work that APC/C remains active in post-mitotic plant cells and that APC/C hypomorphic mutant lines exhibit severe developmental abnormalities such as disorganized vascular tissue, indicating a role for APC/C in plant vasculature development and organization [18]. More recently, Zheng et al. identified APC/C as a dual integrator controlling both Cyclin B1;1 (CYCB1;1) degradation and transcriptional regulation during male gametogenesis [19]. This work highlighted that APC/C is necessary to recruit RNA polymerase II to *MIR159* promoters as in *apc/c* mutants the level of miR159 is reduced which leads to an accumulation of *DUO1* transcripts and therefore of *CYCB1;1* transcripts.

In order to identify new substrates of APC/C, we performed a yeast two-hybrid screen using APC10/DOC1 subunit as a bait. Among all putative interactors that were obtained, we focused our attention on a protein, which belongs to the Double-stranded RNA Binding protein (DRB) family, named DRB4. DRB proteins associate with Dicer-like proteins (DCLs) and are involved, for instance, in the biogenesis of miRNA or trans-acting siRNA (tasiRNA) in plants [20], [21], [22]. DRB4 contains two dsRNA binding motifs (dsRBD1 and 2) in its N-terminal half [23]. It interacts with DCL4, one of the four Dicer-like proteins present in *Arabidopsis*, to generate 21 nt-long sRNAs (such as tasiRNA, DCL4-dependent miRNAs or viral derived siRNAs) from endogenous or exogenous dsRNAs [21], [22], [24], [25], [26]. In a recent work, DRB4, and more precisely its dsRBD2 motif, was shown to be essential for DCL4 activity *in vitro* [27]. Here, we show that APC10 specifically interacts with DRB4, that both genes have similar expression profiles and that DRB4 protein overaccumulates in *apc10* hypomorphic mutants. All together this work provides novel insights into APC/C functions outside of the cell cycle, and connects two important regulatory pathways, selective protein degradation and RNA silencing.

## Results

### The APC10 subunit of the APC/C complex physically and specifically interacts with DRB4

**Figure 1:**
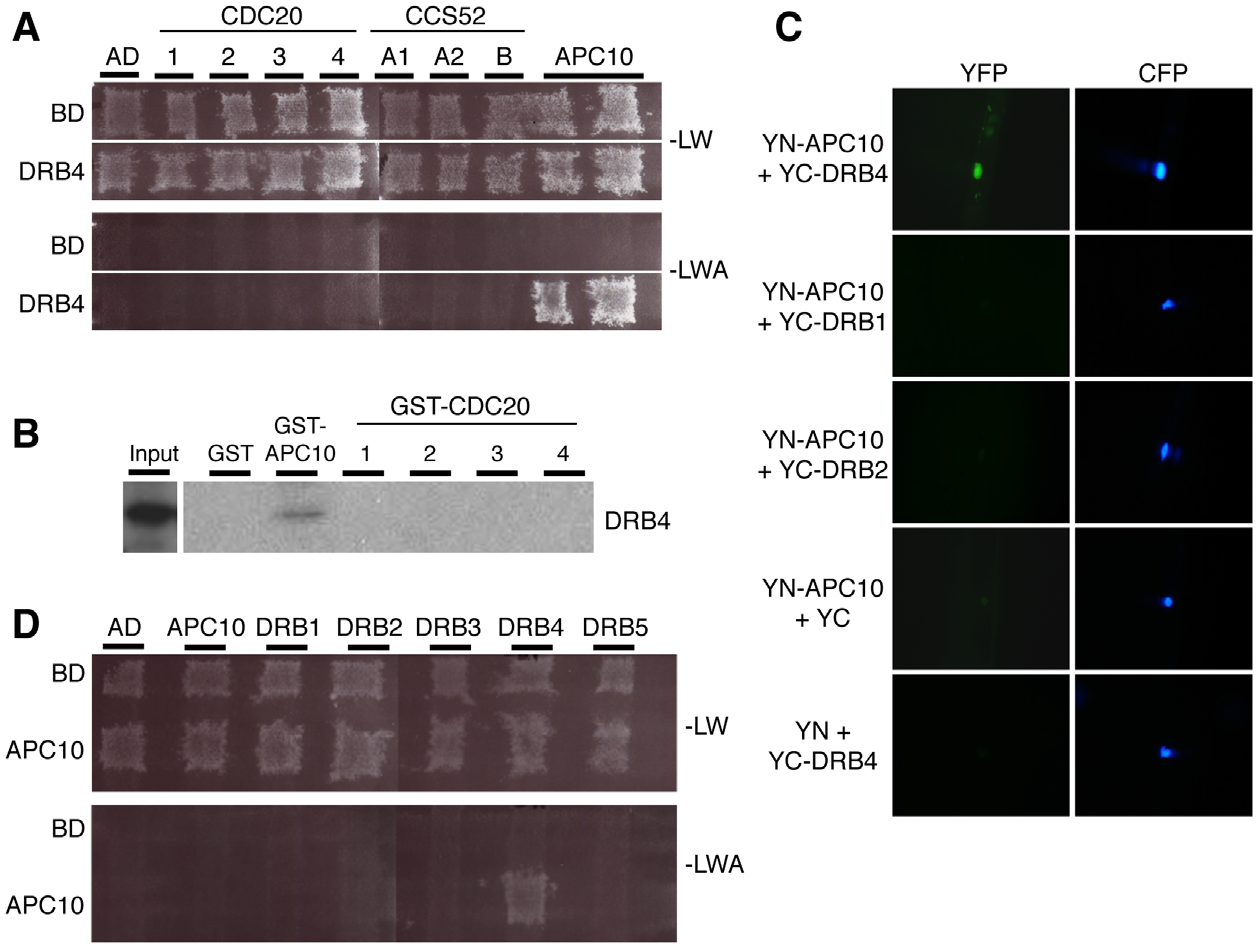
Specific interaction between APC10 and DRB4 occurs *in vitro* and *in vivo*. (**A**) Yeast two-hybrid analyses were performed by mating on non-selective (-LW) and selective (-LWA) media. DRB4 was fused to the binding-domain (BD) whereas APC10, CDC20-1/-2/-3/-4 and CCS52A1/A2/B were fused to the activation domain (AD). Empty BD and AD vectors were used as negative controls. (**B**) [^35^S]methionine-labelled DRB4 was incubated with recombinant GST-APC10, GST-CDC20-1/-2/-3 or GST alone. After several washes, proteins were affinity-purified on glutathione-Sepharose beads, and loaded on an acrylamide gel. The pulled-down proteins were analyzed by autoradiography. The same result was obtained in three independent experiments. (**C**) Bimolecular fluorescence complementation showed APC10 and DRB4 interaction *in planta*. Recombinant YN-APC10 and YC-DRB1/2 or 4 were co-bombarded together with a NLS-CFP construct into 4 day-old mustard seedlings. Fluorescence was observed using an E800 fluorescence microscope. YN and YC alone were used as negative controls. Reconstitution of functional YFP as detected by YFP fluorescence occurs only in the nucleus. A strong YFP signal was observed in the nucleus of 91% of examined cells (64/70). No fluorescence signal was obtained after bombardment with the following plasmid combinations YN-+YC-DRB4 and YN-APC10+YC- or with YN-APC10+YC-DRB1 and YN-APC10+YC-DRB2. (**D**) Yeast two-hybrid analyses were performed by mating on non-selective (-LW) and selective (-LWA) media. APC10 was fused to the BD and DRB1/2/3/4 and 5 were fused to the AD. Empty BD and AD vectors were used as negative controls.

In order to identify novel APC/C substrates, a yeast two-hybrid screen was performed using APC10/DOC1 as the bait to screen a three week-old *Arabidopsis* seedlings library. Among 134 clones that were obtained, forty-two were re-confirmed as putative interactors after plasmid retransformation. Among them, the clone API2 (APC10 Interactor 2) was fished nine times in this screen and sequencing revealed that its cDNA corresponds to the At3g62800 gene encoding DRB4 (for Double-stranded RNA Binding protein 4). The full-length cDNA of DRB4 was amplified and cloned into the yeast two-hybrid vector allowing us to further confirm the interaction with APC10 (Figure 1A). To validate this interaction obtained in yeast, we performed *in vitro* pull down assays. For this purpose, we generated a GST protein-fusion with APC10 that was expressed in *E. coli* and subsequently purified. The DRB4 protein labelled with [^35^S] methionine was produced *in vitro*. These assays showed that DRB4 was pulled down with GST-APC10, but not with GST alone (Figure 1B). Finally, we also validated this protein interaction *in planta* by bimolecular fluorescence complementation (BiFC) experiments. Plasmids YN-APC10 and YC-DRB4 were co-bombarded into etiolated mustard hypocotyls, and a strong YFP signal was observed in the nucleus of examined cells (Figure 1C).

APC10 is a core APC/C subunit that participates in the recruitment of substrates, though this is mainly achieved by APC/C co-factors belonging to the CDC20/FIZZY or CDH1/FIZZY-RELATED families [4]. In *Arabidopsis*, six *CDC20* genes and three *CDH1* genes (called *CCS52A1*, *CCS52A2* and *CCS52B*) have been predicted [14], and a recent study has demonstrated that in the CDC20 family only two isoforms, AtCDC20.1 and AtCDC20.2, seem to be functional [28]. We then tested by yeast two-hybrid and GST pull-down assays whether DRB4 interacts with these different co-factors. From this analysis, we conclude that DRB4 only interacts with APC10, but not with any of the other co-factor tested (Figure 1A and B). On the other hand, DRB4 belongs to a small multigenic family with five members in *Arabidopsis*, named DRB1 to 5 [23]. Therefore we tested whether APC10 is also able to interact with other members of this protein family. Using the yeast two-hybrid assay and BiFC, we could only detect an interaction between APC10 and DRB4 (Figure 1C and D, and data not shown). Moreover, this interaction was localized in the nucleus, in agreement with previously published sub-cellular localizations for DRB4 and APC10 [23], [29]. From these results we conclude that the interaction between APC10 and DRB4 is specific and occurs in the nuclei of plant cells.

### The second dsRNA binding domain of DRB4 is necessary for its interaction with APC10

**Figure 2:**
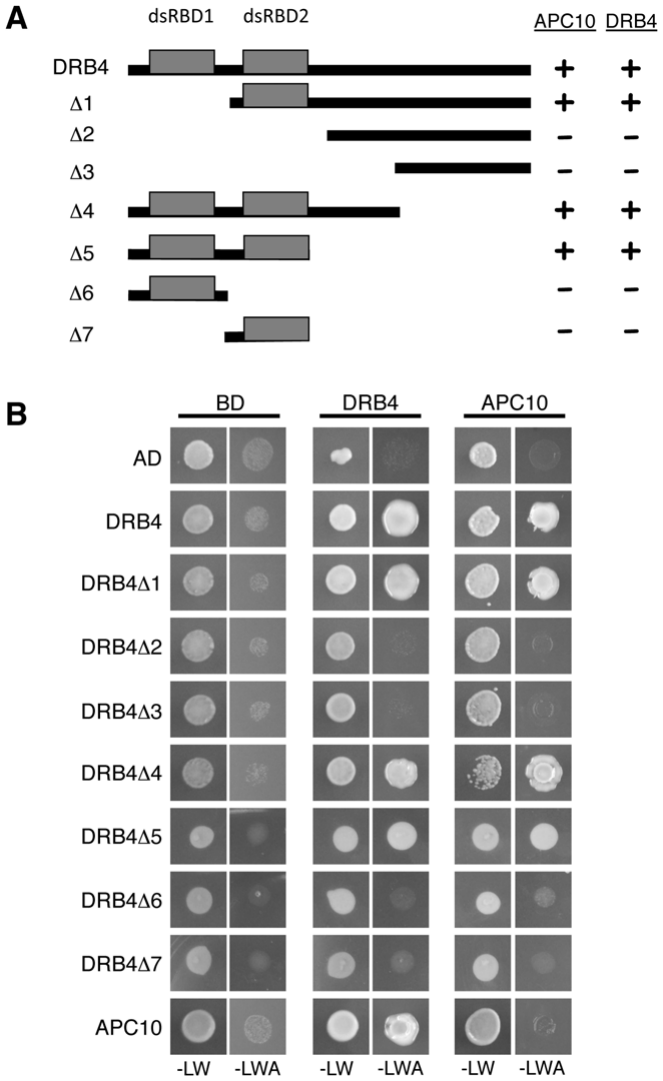
Mapping of interaction domains between APC10 and DRB4. (**A**) Schematic representation of DRB4 deletion constructs generated and fused to the activation domain (AD). Interaction with full-length APC10 or DRB4 fused to the binding-domain (BD) was then scored by yeast two-hybrid assays (see B). A summary of three independent assays is indicated on the right. +, interactions scored based on growth on selective media. −, no growth on selective media. (**B**) One of the three yeast two-hybrid assays between DRB4 or APC10 and DRB4 deleted versions. After mating, yeast was grown at 28°C for 3 days on non-selective (−LW) or strong selection (−LWA) media. Empty AD and BD vectors were included as negative controls.

To further understand how APC10 interacts with DRB4, we generated deleted versions of DRB4 that were tested in yeast two-hybrid assays. First, we observed that DRB4 full-length is able to dimerize (Figure 2). Moreover, our results showed that the same domain of DRB4 that is responsible for its dimerization is also required for its interaction with APC10. Thus the interaction between both proteins is lost with the deleted versions Δ2 and Δ3 of DRB4, which lack the two dsRBDs, but is maintained when only the first dsRBD is absent (deletion Δ1). This suggests that the second dsRBD is necessary for this interaction. However, this domain alone (deletion Δ7) does not interact with DRB4 or APC10, suggesting that dsRBD2 is necessary but not sufficient for either DRB4 dimerization or its interaction with APC10.

### APC10 and DRB4 exhibit similar expression patterns

To further investigate the relevance of the APC10-DRB4 interaction, we compared the expression profiles of both genes in different plant tissues. It was previously described by using GUS reporter lines, that DRB4 was specifically expressed in the vasculature, the root and the shoot apical meristem [30]. Thus, we generated *Arabidopsis* transgenic lines expressing the *GUS* gene under the control of the *APC10* promoter region. A 1500 bp region upstream of the ATG in addition to the first exon, the first intron and the beginning of the second exon of *APC10* was cloned in frame with the *GUS* reporter gene. Stable transgenic lines were generated and twenty independent lines were selected for a unique copy of the transgene. Five representative lines were further analyzed in details. Staining for detection of GUS activity revealed a strong expression in vascular tissues in leaves, hypocotyls and roots, and in the shoot apical meristem (Figure S1). Surprisingly for a gene involved in cell cycle function, its expression was not detected in meristematic tissue within the apical region of root tips. However, this observation is in agreement with a recent expression analysis of *APC10* [29], and is also consistent with the expression profile of *APC6*, another APC/C subunit [31]. Our results together with published data show that APC10 and DRB4 exhibit overlapping expression profiles.

### DRB4 is a novel substrate of the APC/C complex

**Figure 3:**
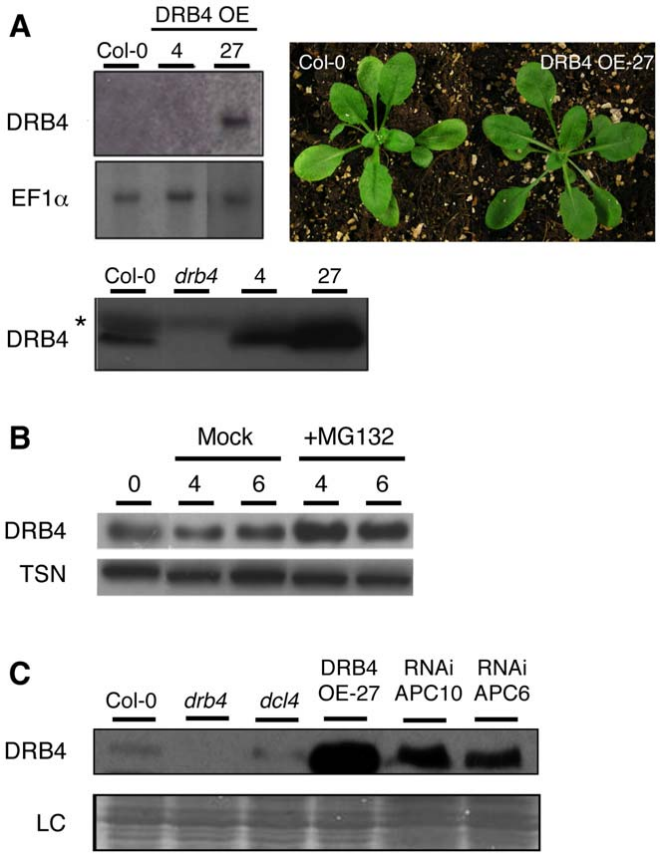
DRB4 over-accumulates after MG132 treatment and in APC/C RNAi lines. (**A**) Characterization of DRB4 overexpressing lines. Northern blot analysis (upper panels) of *DRB4* mRNA accumulation in flower extracts from Col-0 and two independent transgenic lines expressing DRB4 under the control of the strong 35S promoter (referred hereafter as lines DRB4 OE-4 and -27). Accumulation of EF1α mRNA is used as a loading control. Western blot analysis (lower panel) performed using an antibody directed against DRB4. The asterisk indicates a non-specific cross-reacting band that can also be used as a loading control. Pictures of 5 week-old Col-0 and DRB4 OE-27 plants. (**B**) Three week-old DRB4 OE-27 seedlings were incubated in liquid medium supplemented or not with 100 µM MG132. After 4 h and 6 h of incubation, seedlings were collected and total protein extracted. Western blot was performed using antibodies against DRB4 or TSN as a loading control. (**C**) Western blot analysis of DRB4 accumulation in flower extracts from Col-0, *drb4*, *dcl4*, DRB4 OE-27 line, RNAi APC10-38 and RNAi APC6-20 lines. Coomassie staining was used as a loading control (LC).

*DRB4* loss-of-function mutations exhibit a zippy phenotype with elongated and downward curled leaves due to reduced accumulation of *TAS3* tasiRNAs [21], [22]. First, we decided to generate *Arabidopsis* lines that over-accumulate DRB4 protein to evaluate whether this would also compromise normal plant development. The full-length cDNA of DRB4 was cloned downstream of the CaMV 35S promoter and *Arabidopsis* plants were transformed with this construct. Thirty independent lines were selected and analyzed at the molecular level for *DRB4* expression. Figure 3A presents the results of two independent lines, accumulating either somewhat similar (line DRB4 OE-4) or strongly increased (line DRB4 OE-27) DRB4 levels in comparison to wild-type control plants. It is noteworthy that endogenous DRB4 transcripts are difficult to detect on RNA gels. In contrast with *drb4* null mutation, over accumulation of DRB4 did not produce any obvious phenotypic alterations compared to wild type during the whole plant development (Figure 3A and data not shown).

If DRB4 is a substrate of the APC/C, it should be degraded by the 26S proteasome. Endogenous DRB4 protein can easily be detected in plant inflorescences (Figure 3A), but not in seedlings, a plant material in which the proteasome activity can be pharmacologically inhibited. Therefore we used the DRB4 OE-27 line to treat three week-old seedlings with the proteasome inhibitor MG132 and samples were collected after 4 and 6 hours of treatment. Total proteins were extracted and western blots were performed using antibodies against DRB4 and RNA binding protein Tudor-SN (TSN) as a loading control (Figure 3B). The increase of DRB4 protein accumulation observed in MG132-treated samples, but not in mock-treated seedlings, suggests that DRB4 protein is degraded by the 26S proteasome.

Based on the above result, we reasoned that plants defective for APC/C activity should accumulate more DRB4 protein than wild-type plants. Loss-of-function mutations in APC/C subunits lead to early arrest during gametogenesis in *Arabidopsis*, therefore to test our hypothesis, we used our previously established hypomorphic RNAi lines for two APC/C subunits, APC6 and APC10, showing reduced APC/C activity [18]. Western blot analysis performed on flower extracts revealed an over-accumulation of DRB4 in both APC10 and APC6 hypomorphic RNAi lines compare to wild-type plants. This increased accumulation of DRB4 in genetic backgrounds where APC/C activity is reduced strongly suggests that DRB4 is indeed a genuine new target of the APC/C.

### DRB4 over-accumulation does not affect the siRNA pathway

**Figure 4:**
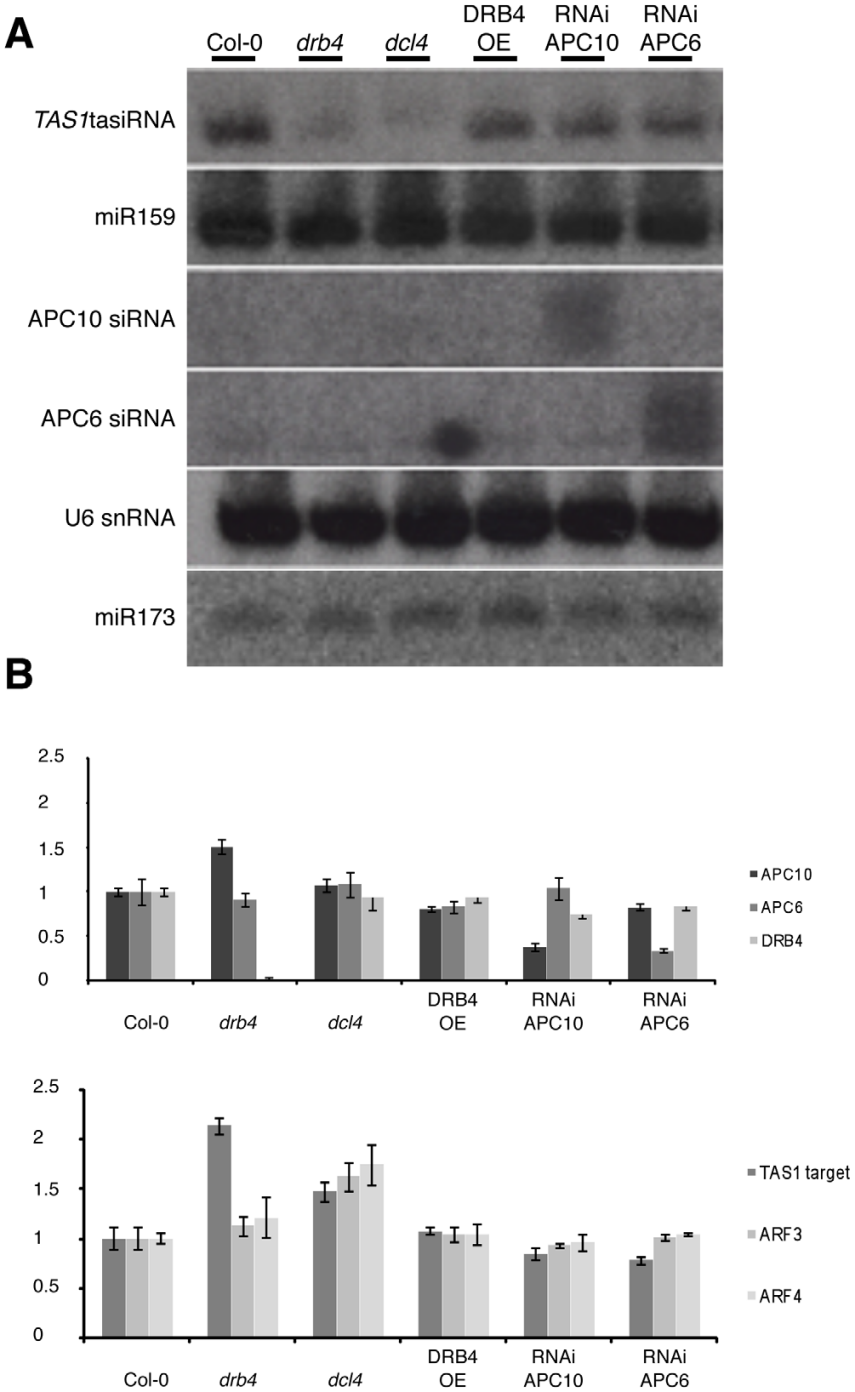
Over-accumulation of DRB4 does not affect *trans*-acting siRNA biogenesis and their targets accumulation. (**A**) Northern blot analysis of various sRNA accumulation in Col-0, *drb4*, *dcl4*, DRB4 OE27, RNAi APC10-38 and RNAi APC6-20 lines. *Trans*-acting siRNA and miRNA accumulation were detected using a *TAS1* tasiRNA255, a miR159 specific probe and a miR173 specific probe, respectively. APC10 and APC6 probes were used to score the accumulation of siRNA targeted against APC10 and APC6 genes in their respective RNAi lines. U6 was used for the loading control. (**B**) Quantitative real-time PCR reactions were performed on total RNA from the same background depicted in (A). Specific primers against *APC10*, *APC6*, *DRB4* and three target genes of the tasiRNA pathway (*TAS1* target, *ARF3* and *ARF4*) were used. RNA levels were normalized to that of Actin2 (At3g18780) and then to the value of the wild-type plants, which was arbitrarily set to 1. Error bars represent standard deviation from 2 independent experiments involving triplicate PCR reactions each.

In the tasiRNA pathway, miRNA-loaded RISC mediate the initial cut of non-coding *TAS* precursor transcripts [32], [33], [34], [35]. This, in turn, promotes the tasiRNA biogenesis cascade through conversion by RDR6 of the cleaved precursors into long-dsRNA that is processed by DCL4 into 21 nt-long tasiRNAs in a process that also requires DRB4 [32], [36], [37], [38], [39]. Consequently, tasiRNA targets over-accumulate in both *dcl4* and *drb4* mutants [22]. Therefore, we decided to test the accumulation of tasiRNA and their targets in the DRB4 OE line and APC/C hypomorphic lines that also over-accumulate DRB4, as well as miR173, which is the miRNA triggering the synthesis of *TAS1* tasiRNA. However, we could not detect a difference in the accumulation of *TAS1* tasiRNA (Figure 4A), miR173 (Figure 4A) or *TAS3* tasiRNA (data not shown) in the DRB4 OE-27 line nor in APC/C hypomorphic lines compared to wild type plants, whereas 21-nt tasiRNA level was significantly reduced in *drb4* and absent in *dcl4* mutants (Figure 4A). Probing for miR159 accumulation revealed that the DCL1-dependent miRNA pathway was also not affected in any of the tested mutants (Figure 4A). It is also known that some miRNAs derive from DCL4-mediated processing of pri-miRNA precursors [40]. We therefore tested the accumulation of miR822 – a DCL4-dependent miRNA - in our different backgrounds. We were not able to detect any difference in the accumulation of miR822 in the DRB4 OE-27 line nor in APC/C hypomorphic lines compared to wild type plants (data not shown).

Using quantitative-PCR (qPCR), we also analyzed the accumulation of *TAS1* and *TAS3* targets in the different genetic backgrounds (Figure 4B). The upper panel shows the level of endogenous APC10, APC6 and DRB4 transcripts in the different lines. The levels of *APC10* and *APC6* are reduced in their respective hypomorphic lines, whereas the transcript level of *DRB4* is unchanged in these lines. With the exception of a slight but significant increase of transcript level for TAS targets in *drb4* and *dcl4* mutants, no significant difference was observed in DRB4 OE-27 and APC/C hypomorphic lines. Therefore, from these experiments we can conclude that over-accumulation of DRB4 does not affect the tasiRNA pathway.

### Viral infection is not affected by DRB4 over-accumulation

The antiviral RNA silencing pathway also requires DCL4 and DRB4 for production of viral-derived siRNA (vsiRNA). It was previously shown that in *drb4* and *dcl4* mutants, accumulation of those 21-nt vsiRNAs were reduced or absent, respectively, and that, concomitantly, accumulation of 22-nt and 24-nt vsiRNAs was strongly increased [30]. To test the effect of DRB4 over-accumulation on viral infection, DRB4 OE lines were infected with modified Tobacco rattle virus (TRV-PDS) where the RNA2-encoded 2b and 2c sequences were replaced by a fragment of the *Arabidopsis* phytoene desaturase (PDS) gene. This virus triggers the appearance of a strong photobleaching phenotype in infected tissues due to virus-induced gene silencing (VIGS) of the PDS [24].

**Figure 5:**
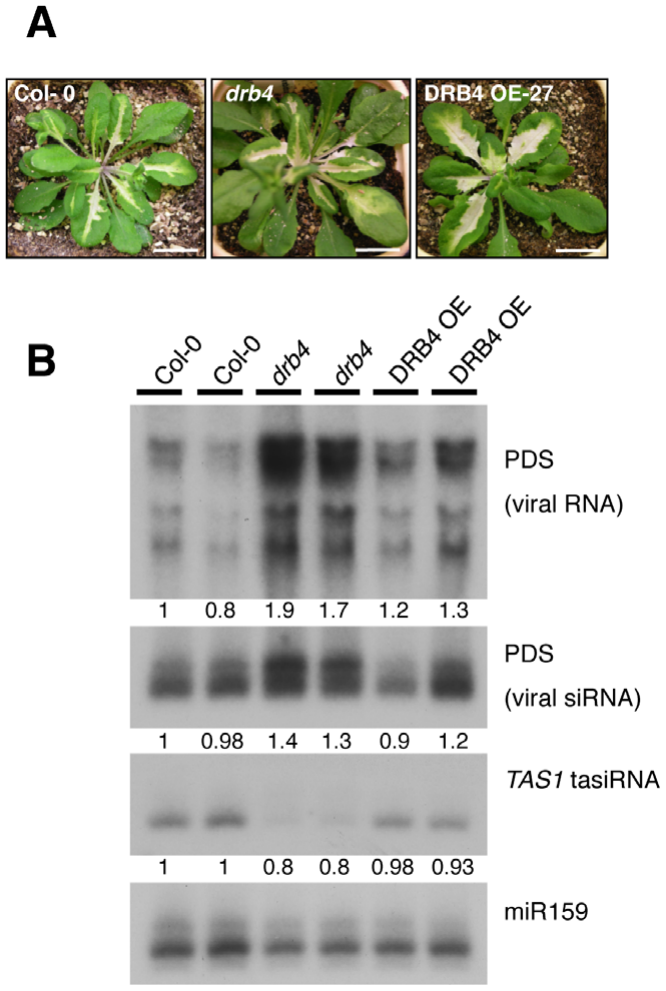
Viral infection of *drb4* and DRB4 OE line. (**A**) Pictures from infected plants with the TRV-PDS virus, 14 days post-infection (dpi). Scale bar: 1 cm. (**B**) Northern blot analysis of TRV-PDS viral RNA or viral-derived siRNA accumulation in Col-0, *drb4* and DRB4 OE-27 line using a PDS specific probe. TAS1 tasiRNA accumulation was detected using a siRNA255 probe. miR159 accumulation was used here as a loading control. Values are normalized to miR159 and are expressed as a ratio relative to the wild-type Col-0, which was arbitrarily set to 1.

Figure 5A illustrates the photobleaching phenotype in wild type, *drb4* and DRB4 OE plants 14 days post-inoculation. The extent and consistency of VIGS was not altered in DRB4 OE lines compared to WT- or *drb4*-infected plants. Northern blot analysis was then performed on RNA extracted from infected leaves using a PDS probe to detect viral RNA and vsiRNA (Figure 5B, the two upper panels, respectively). As expected, increased levels of viral RNA were detected in *drb4*. However, in plants over-accumulating DRB4, viral RNA accumulation and vsiRNA patterns were similar to those detected in wild-type plants suggesting that overexpression of DRB4 does not significantly affect the antiviral RNA silencing pathway.

### Effects of DRB4 over-accumulation on heterochromatic siRNAs

**Figure 6:**
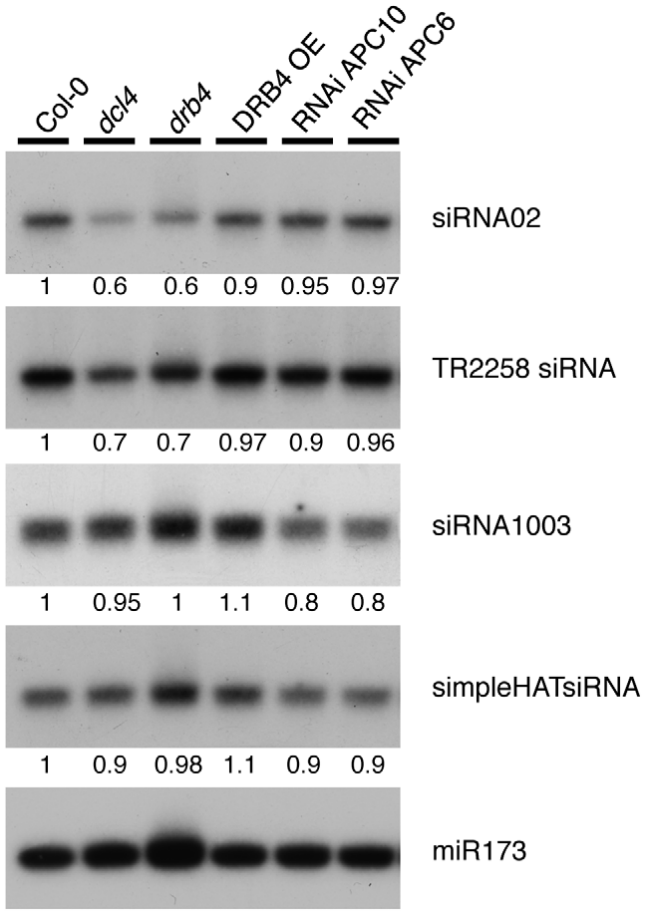
Reduced APC/C activity slightly affect polIV/polV-dependent heterochromatic siRNA accumulation. Northern blot analysis of polIV-dependent (siRNA02, TR2258) or polIV/polV-dependent (siRNA1003, simpleHAT) siRNA accumulation in Col-0, *drb4*, *dcl4*, DRB4 OE27, RNAi APC10-38 and RNAi APC6-20 lines. miR173 accumulation is used here as a loading control. Values are normalized to miR173 and are expressed as a ratio relative to the wild-type Col-0, which was arbitrarily set to 1.

It was recently published that DRB2 and DRB4 have antagonistic impact on RNA polymerase IV (polIV)-dependent siRNA (p4-siRNAs) levels [40]. Indeed, loss of DRB2 resulted in increased accumulation of p4-siRNAs, whereas *drb4* mutant exhibited reduced p4-siRNAs levels, although the extent of this reduction was variable. Moreover, transgenic plants overexpressing DRB2 mimicked *drb4* mutants, and exhibited reduced p4-siRNA levels. Based on those findings, we decided to monitor the accumulation of a set of 24 nt heterochromatic siRNAs in our hypomorphic APC10 or APC6 lines together with the DRB4-overexpressing plants. In agreement with previous results [40], TR2258 and siRNA02, two 24 nt-long siRNAs produced from polIV-dependent loci [41] were down-regulated in *dcl4* and *drb4* mutants (Figure 6). However, their accumulation in our transgenic lines was not affected compared to wild type plants suggesting that over-accumulation of DRB4 does not impact p4-siRNAs levels. We next checked the accumulation of 24 nt siRNAs SimpleHAT2 and siRNA1003, two sRNAs that require, in addition to polIV, the activity of RNA polymerase V (polV) for their production [41], [42]. Interestingly, these polIV/polV-dependent siRNAs were not affected by *dcl4* or *drb4* mutations but showed a slight but consistent reduction in hypomorphic APC10 or APC6 lines (Figure 6). However, as this decrease in their accumulation was not observed in our DRB4 overexpressing line, it suggests that this apparent down-regulation is not directly related to the increased levels of DRB4 in APC10/APC6 hypomorphic mutants.

## Discussion

### DRB4 is the first non-cell cycle APC/C complex substrate in plants

For many years, the APC/C complex was only known as the major E3 ubiquitin ligase regulating the cell cycle. It is just only recently that new functions of APC/C have been described in the organization, size and growth of differentiated tissues and cells, such as neurons [9], [11], [12], [13]. In these processes several new APC/C targets have been identified. Here, we describe for the first time a substrate of the plant APC/C which is not involved in the regulation of cell cycle. Our results have shown that DRB4 interacts specifically with APC10, is able to homodimerize and that the 2^nd^ dsRBD is necessary for this interaction as well as for DRB4 homodimerization. Interestingly, this domain seems to be very important for DRB4 function as it is essential for dsRNA binding and for DCL4 interaction and activity [27]. By western blot assays it was also shown that DRB1 (also known as HYL1) is able to homodimerize and even to heterodimerize with other DRB proteins, though these interactions were weaker than with DCL1 [23]. Moreover, a recent study has highlighted an antagonistic role of DRB2 and DRB4 on RNA polymerase IV-dependent siRNA levels [40]. Hence, it was shown that in *drb2* mutant the level of p4-siRNA is significantly increased, whereas in *drb4* or in a DRB2 over-accumulating line this level is reduced. These results support that DRB proteins are part of multiple protein complexes with different functions. Moreover, maintaining proper DRB proteins accumulation might be of importance as these proteins, through homodimerization or heterodimerization, could compete for binding to dicer proteins and thus modulate their activity.

In plants, the APC/C has only been involved in cell cycle regulation and endoreduplication [15], [16], [17], [18], [43], [44], [45]. Here, we identified a new substrate of the APC/C complex, which is not involved in the regulation of the cell cycle but in siRNA biogenesis pathways. A recent work has also shown a link between APC/C and miRNA biogenesis pathway during male gametophyte development [19]. Analyses of *apc8-1* and *apc13-2* mutants revealed that APC/C was necessary for tricellular pollen development and that it was involved in the recruitment of RNA polymerase II to the miR159 promoter. In fact, the level of miR159 was reduced in these *apc* mutants leading to an increase in *DUO1* expression. DUO1 being a transcription factor activating *CYCB1;1*, they observed an increased expression of this cyclin in these mutants, which defines the APC/C complex as a factor regulating both transcription and degradation of CYCB1;1. Though it is still unclear if APC/C acts directly or indirectly on the Pol II recruitment to miR159 promoter, this work established a role of the APC/C in sRNA biogenesis and most likely at a very early step of the pathway. While in this study a clear reduction of miR159 in *apc* mutants was observed, in our case we could not detect any change in the miR159 level between wild type and *APC* hypomorphic lines (Figure 4). This discrepancy can be explained by the fact that we used different tissues to analyze sRNA accumulation and that the effect of APC/C on miR159 might be specific to pollen development.

### Over-accumulation of DRB4 does not alter plant development

Our results revealed a strong over-accumulation of DRB4 protein in APC hypomorphic mutants. These mutants exhibit a number of developmental alterations [18], which could however be attributed to a failure to degrade many other proteins beside DRB4. Indeed, DRB4 protein over-accumulation in transgenic plants did not result in any obvious phenotype compared to wild type during plant development nor at a molecular level on tasiRNA accumulation. This result is even more surprising, as it was showed that DRB2 over-expressing lines show a phenotype that copies a *drb4* mutant at molecular and morphological levels [40]. One possibility to explain the lack of phenotype in DRB4 OE lines is that in the tasiRNA pathway, different steps involve different actors which could also be limiting factors. For instance, DRB4 interacts with DCL4 to cleave long dsRNA into 21-nt small siRNAs. Thus, though the level of DRB4 is increased, if this is not also the case for DCL4 and/or its RNA targets, it may not affect sRNA production. However, we cannot exclude the possibility that the regulation of DRB4 protein turnover by the APC/C may become important in specific conditions such as in response to some stresses or environmental changes.

### The UPS: another level of control for the sRNA biogenesis pathway

Intensive research on the RNA silencing field over the past decade has brought to light the existence of several layers of regulation on its machinery [46]. Additionally to transcriptional regulation, several studies have shown that both sRNAs and their associated proteins can be posttranscriptionally or posttranslationally modified at multiple steps of the pathway. The 3′-end of sRNA are highly heterogeneous and has been shown to be subjected to various modifications. Thus, the *Arabidopsis* methyl transferase, HEN1 adds a methyl group to the 2′-OH at the 3′-end of sRNAs which protects them from uridylation and degradation [47]. In mammals, miRNAs have been shown to be adenylated and this modification would affect both their stability and their activity [48].

Proteins associated to sRNA and involved in their biogenesis are also subjected to posttranslational modifications. In human, Drosha, an enzyme required for pri-miRNA processing, was found phosphorylated and that this protein modification was essential for its localization into the nucleus [49]. TRBP is a human dsRNA-binding protein, which has also been shown phosphorylated [50]. This modification enhances TRBP stability and consequently increases the level of its associated protein Dicer. However, in our DRB4 over-accumulating plants, we failed to detect a significant increase in the level of its associated dicer, DCL4 (data not shown). This indicates that an over-accumulation of the dsRNA-binding protein does not necessarily lead to an over-accumulation of its dicer partner. Argonaute2 (Ago2) is another player of the sRNA pathway found to be modified. Human Ago2 can be hydroxylated at a proline residue, which has a role on its stability and its localization to the processing bodies [46]. Moreover it has been shown that mouse Ago2 is ubiquitylated and targeted for degradation by the 26S proteasome [51]. This process is mediated by Lin41, a stem cell-specific E3 ubiquitin ligase that directly interacts with Ago2. In our study, we revealed another connection between the UPS and the sRNA regulatory pathways by showing that a player of the sRNA biogenesis is regulated through this degradation machinery. While we cannot exclude that the APC/C-dependent degradation of DRB4 has some regulatory roles under specific growth conditions, it is also conceivable that this E3 ubiquitin ligase has some basic housekeeping functions in maintaining precise cellular-protein concentrations and homeostasis of key regulatory proteins.

## Materials and Methods

### Constructs and primers

Full-length cDNA from APC10, CDC20-1 to -4, CDH1.1 to 1.3 and DRB1 to 5 were amplified and cloned into a Gateway™ entry vector. cDNAs were then cloned into different destination vectors according to the interaction assay. The pGAD-T7-GW and pGBT9-GW (Clonetech and modified with the Gateway™ technology from Invitrogen) were used for yeast two-hybrid screen and pair-wise interactions, the pDEST15 (Invitrogen) was used for GST pull-down assay, and the pMAV-YN-GW and pMAV-YC-GW [52] were used for BiFC assay. The deleted versions of DRB4 were also cloned into a Gateway™ entry vector and then transferred by recombination into yeast two-hybrid vectors for the interaction tests. The primers used to generate the deletions are the following: deletion 1 (GGATCCGAGGGAATTGATGTTGCC/GATATCTTATGGCTTCACAAGACG), deletion 2 (GGATCCTCGAACCAGACCGGA/GATATCTTATGGCTTCACAAGACG), deletion 3 (GGATCCGGTATGAAGATGAACATTGC/GATATCTTATGGCTTCACAAGACG), deletion 4 (GGATCCATGGATCATGTATACAAAGGTC/GATATCATCAGTAGTTAGTGCACTAAG), deletion 5 (GGATCCATGGATCATGTATACAAAGGTC/GATATCGTTCCCATTTTTGATATCTCATG), deletion 6 (GGATCCATGGATCATGTATACAAAGGTC/TGGACTTTGTGGCGTCAA) and deletion 7 (GGATCCGAGGGAATTGATGTTGCC/GATATCGTTCCCATTTTTGATATCTCATG).

### APC10 promoter analysis

A fragment containing 1.5 kb upstream of the ATG, the first exon, the first intron and the begining of the second exon of APC10 gene was cloned into pMDC163 vector [53] to trigger the expression of the βGlucuronidase (GUS) reporter gene. *Arabidopsis* plants were stably transformed using the ‘floral dip’ method [54] and 20 independent lines were selected for a unique copy of the transgene.

### DRB4 over-expressing line and other mutant lines

DRB4 full-length cDNA was cloned by LR recombination into pK2GW7 vector [55] which allows its expression under the control of the CaMV 35S promoter. *Arabidopsis* plants were stably transformed using ‘floral dip’ method [54] and 30 independent lines were selected for a unique copy of the transgene. *drb4-1* and *dcl4-2* mutants were previously described [21], [36]. The generation and molecular characterization of *APC10* and *APC6* hypomorphic lines (RNAi APC10 and RNAi APC6) were previously published [18].

### Yeast two-hybrid assays

Yeast two-hybrid assays were performed using *Saccharomyces cerevisiae* haploid strains PJ69-4a and PJ69-4 alpha (*MATa/alpha*, *trp1-901*, *leu2-3*, −*112*, *ura3-52*, *his3-200*, *gal4*Δ, *gal80*Δ, *LYS::GAL1-HIS3*, *GAL2-ADE2*, *met2::GAL7-lacZ*) [56]. The yeast two-hybrid was performed using cross-mating assays, as described in [57]. Diploid yeasts were selected on synthetic defined (SD)/-Leu/-Trp (SD-LW) medium, whereas interactions were selected on SD/-Leu/-Trp/-Ade (-LWA) medium.

### GST Pull-Down

*E. coli* (BL21) was transformed with the different constructs or the empty vector and grown at 37°C until *A*_600_ = 0.5. Synthesis of glutathione *S*-transferase (GST) fusion proteins was induced by addition of 20% Arabinose, and the bacteria were incubated for an additional 4 h at 28°C. Bacteria were harvested, resuspended in extraction buffer (150 mM NaCl/1 mM EDTA/100 mM Tris, pH 7.5/1 mM DTT/0.5% NP40/10 mM β-glycerine phosphate/1 mM NAF/1 mM PMSF) plus a protease inhibitor mixture (Roche), and sonicated. GST-tagged proteins were isolated by affinity chromatography on glutathione-Sepharose beads (GE Healthcare). [^35^S]methionine-labelled DRB4 was produced by coupled *in vitro* transcription and translation of pGBKT7–DRB4 using a TNT-Quik kit (Promega). The translation product was incubated for 4 h at 4°C with the beads. After washing with extraction buffer, immobilized proteins were taken up in gel-loading buffer, and subjected to SDS/PAGE. Total proteins in the gel were visualized by Coomassie blue staining, and ^35^S–DRB4 was identified by autoradiography.

### *In planta* Bimolecular Fluorescence Complementation (BiFC)

Mustard (*Sinapis alba* L.) seeds were sown on four layers of moist filter paper in Plexiglass boxes and cultivated for 4 days in darkness at 25°C. For transformation and BiFC experiments, etiolated seedlings were fixed to standard glass microscope slides with surgical adhesive (B-400 Secure Adhesive; Factor II Inc., Lakeside, AZ, USA). The split-YFP and CFP constructs (5 µg of each plasmid) were introduced into mustard hypocotyl cells with gold particles bombardment, as described in [52]. After transformation, mustard seedlings were placed vertically in sterilized water and kept at 25°C in darkness overnight prior to microscope analysis. Images were recorded with an E800 fluorescence microscope (Nikon) by using CFP- and YFP-specific filters.

### GUS Staining

For GUS staining, the plant material was fixed in 80% acetone on ice for 20 minutes and then washed two times with 0.05 M Phosphate buffer. Plants were then immersed in the enzymatic reaction solution (0.05 M Phosphate Buffer/0.005% Triton/0.5 M ferricyanide/0.5 M ferrocyanide/10 mM EDTA/1 mM 5-bromo-4-chromo-3-indolyl β-d-glucuronide) and incubated overnight at 37°C in the dark. The plant material was cleared with ethanol washes and examined under a light microscope (E800, Nikon).

### MG132 treatment

Seeds from DRB4 OE line were sown on MS medium and grown for three weeks. Seedlings were then transferred into MS liquid medium and a sample was taken and frozen for time 0. Medium was then supplemented or not with MG132 (100 µM) and seedlings were incubated at room temperature for several hours.

### Western Blot

Total protein were extracted from three week-old seedlings or floral buds and analyzed by SDS-PAGE followed by Western blot using 1∶10000 polyclonal DRB4 antibody [22].

### Northern Blot

Total RNA was extracted using TRIzol (Invitrogen), precipitated with isopropanol and redissolved in 50% formamide. Northern analyses of low and high molecular weight RNA were performed with 15 and 5 µg of total RNA, respectively, as described in [58]. Radio-labelled probes for detection of APC10, APC6 and TRV-PDS were made by [α-^32^P]dCTP random priming reactions, whereas DNA oligonucleotides complementary to miRNA (159, 173) or tasiRNAs (255) were end-labelled with [γ-^32^P]ATP [24].

### Quantitative PCR (q-PCR)

Total RNA was extracted from leaves using TRIzol (Invitrogen), precipitated with isopropanol and redissolved in water. To remove traces of genomic DNA in RNA samples, equal amounts of total RNA were treated with DNase (Promega) that was subsequently heat inactivated. cDNAs were synthesized by reverse transcription using High Capacity cDNA reverse Transcription Kit (Applied Biosystems™). The cDNAs were then analyzed by the SYBRH Green-Based Detection system (Applied Biosystems) for real-time qPCR. The following primers were used: APC10 (AGGAGAAGTTTGAAGAACATGGA/TGCACAACAACAATAACTAATCTG), APC6 (GCTTGGCTTACACTTACCATTTGC/AATCAACCCCGTTCTGACATTC), DRB4 (AGTGTCTTCACCATTTCAAAGAGA/CTCTCTCATTCGCATACACTGG), TAS1 target (GACATTGGGCGCATGTGG/AGCCTCAATCACTTCTCCTTTCA), ARF3 (TGGTCCCAAGAGAAGCAGG/TCCACCATCCGAACAAGTG), ARF4 (GCCGCTGAAGATTGTTTTGCTC/AGTAGATGCCTCCTTGGTTGACC).

### Viral Infection

TRV-PDS viral transcripts were amplified by inoculation to *Arabidopsis thaliana* leaves. Sap was subsequently extracted from those leaves at 10 dpi (1 g tissue/1 ml 5 mM NaP PH 7.5) and used to inoculate *Arabidopsis* rosette (about 5 weeks old, prior to bolting). Infected leaves were collected at 14 dpi and subjected to molecular analyses (Northern blots).

## Supporting Information

Figure S1 **APC10 expression profile.** A fragment comprising 1.5 kb of promoter region, the first exon, the first intron and the beginning of the second exon of *APC10* was cloned in frame upstream of the *GUS* reporter gene. Several independent transgenic lines were selected and GUS staining was performed on 20 day-old seedlings. The pictures are representative of the expression profiles observed for most of the lines. Scale bar: 100 µm.
(TIF)
