# Supplementary figures and images for: APC/C-Mediated Degradation of dsRNA-Binding Protein 4 (DRB4) Involved in RNA Silencing
Source: PLoS One. 2012 Apr 24;7(4):e35173. doi: 10.1371/journal.pone.0035173 (PMC3335838; doi:10.1371/journal.pone.0035173)

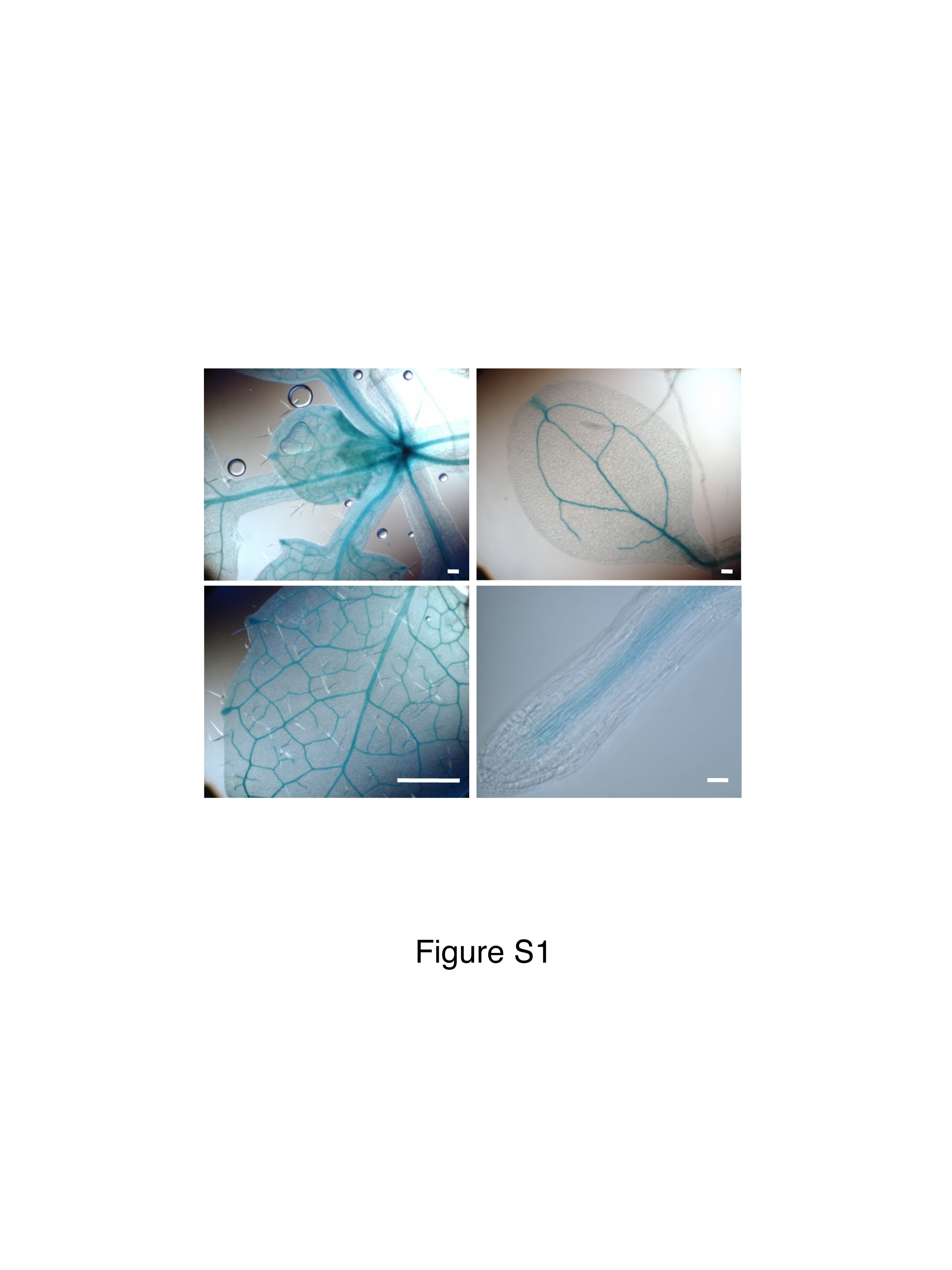

Supplement: Figure S1 — APC10 expression profile. A fragment comprising 1.5 kb of promoter region, the first exon, the first intron and the beginning of the second exon of APC10 was cloned in frame upstream of the GUS reporter gene. Several independent transgenic lines were selected and GUS staining was performed on 20 day-old seedlings. The pictures are representative of the expression profiles observed for most of the lines. Scale bar: 100 µm. (TIF) [file pone.0035173.s001.tif]
